# Effect of artemisinin and neurectomy of pterygoid canal in ovalbumin-induced allergic rhinitis mouse model

**DOI:** 10.1186/s13223-018-0249-6

**Published:** 2018-06-11

**Authors:** Jian Li, Bin Wang, Yingying Luo, Yajie Bian, Ruipei Wang

**Affiliations:** 1 Departments of Otorhinolaryngology and Geriatrics, Cangzhou Central Hospital, 16 Xinhua West Road, Cangzhou, 061000 Hebei People’s Republic of China; 2 Departments of Pediatric Bone Oncology, Cangzhou Combine Traditional Chinese and Western Medicine Hospital, 31 Huanghe West Road, Cangzhou, 061000 Hebei People’s Republic of China; 3 Department of Dermatology, Langfang City Dacheng County Traditional Chinese Medicine Hospital, Cultural Street, Langfang, 065900 Hebei People’s Republic of China; 4 Department of Otorhinolaryngology and Geriatrics, Langfang City Dacheng County Hospital, 47 Xinhua East Street, Langfang, 065900 Hebei People’s Republic of China

**Keywords:** Artemisinin, Allergic rhinitis model, Neurectomy of pterygoid, ERK, Treg cell

## Abstract

**Background:**

Allergic rhinitis (AR), characterized by sneezing, nasal itching and rhinorrhea, affects a large number of population. This study aimed to explore the effects of artemisinin alone or combined with neurectomy of pterygoid canal in ovalbumin-induced AR mouse model and illustrate the underlying mechanisms.

**Methods:**

Allergic symptoms were evaluated to verify inhibitory effect of artemisinin alone or combined with neurectomy of pterygoid canal on AR. Serum levels of histamine, immunoglobulin E (IgE) and inflammatory factors TNF, INF-γ, IL-1β IL-10, IL-4 and IL-5 were measured by ELISA. The mRNA levels of TNF, INF-γ, IL-1β and IL-10 in local lymph nodes were measured by RT-qPCR. The total and phosphorylated levels of ERK and JNK were assessed by Western blot. CD4^+^CD25^+^Foxp3^+^ T (Treg) cells were analyzed by flow cytometry.

**Results:**

Artemisinin significantly relieved the behavior symptoms of AR mice. The administration of artemisinin strikingly suppressed the expression of histamine, IgE and inflammatory factors. An increased Treg cell proportion and inhibited ERK phosphorylation were observed in artemisinin-treated groups as compared to those in the AR group. Moreover, artemisinin plus neurectomy of pterygoid almost abolished the behavioral score increase in AR mice.

**Conclusions:**

These results indicated that artemisinin exhibited anti-allergic effect by inhibiting ERK activation and increasing Treg cell proportion, which subsequently decreased the expressions of allergic mediators. In addition, artemisinin combined with neurectomy of pterygoid showed better efficacy than artemisinin alone.

**Electronic supplementary material:**

The online version of this article (10.1186/s13223-018-0249-6) contains supplementary material, which is available to authorized users.

## Background

Allergic rhinitis (AR), a nasal mucosal inflammation resulting from immunoglobulin E (IgE)-mediated hypersensitivity reaction, is characterized by sneezing, nasal itching, nasal obstruction, in any combination [1, 2]. AR has become a global health problem and affects a large number of population [3]. Allergen exposures induce the recruitment of inflammatory cells, including eosinophils, basophils, mononuclear cells and mast cells, into the nasal mucosa. The activated inflammatory cells release several allergic mediators, such as histamine, leukotrienes and a number of cytokines and chemokines, to sustain inflammatory reactions and generate characteristic nasal symptoms [4]. AR animal models have been studied to illustrate the underlying mechanisms leading to the generation of allergic inflammation and the efficacy of anti-allergic drugs. Repeated ovalbumin (OVA) exposure of mice has been used to establish an AR model with the infiltration of inflammatory cells and increased epithelial layer thickness [2].

Previous studies have reported that some natural plant products, such as Piperine, Citrus sunki and *Zataria multiflora*, possess anti-allergic effect on AR in animal models [2, 5, 6]. The principal sources of biologically active substances are plant natural products. Thus, plant-derived natural products with anti-allergic effect have become much more attracting for research. Artemisinin, an active compound of a traditional Chinese herbal medicine, is an important frontline drug against plasmodium falciparum malaria [7, 8]. Beyond its well-documented anti-malarial property, artemisinin and its derivatives have previously been demonstrated to possess anti-cancer activity both in vitro and in vivo [9–12]. In addition, previous studies have shown that artemisinin possesses anti-inflammatory and anti-fibrosis effects [13, 14].

However, no study has yet been done to examine the possible protective effects of artemisinin on AR. Therefore, we hypothesized that artemisinin treatment might protect against OVA-induced AR. To confirm the above hypothesis, we treated the AR animal model with different doses of artemisinin, and the results showed that artemisinin significantly relieved the behavior symptoms of AR mice. The administration of artemisinin strikingly suppressed the expression of histamine, OVA-specific IgE and inflammatory factors including TNF, INF-γ, IL-1β, IL-10, IL-4 and IL-5. Increased Treg cell proportion and inhibited ERK phosphorylation were found in artemisinin-treated groups as compared to those in the AR group. Moreover, artemisinin combined with neurectomy of pterygoid almost abolished the behavioral score increase in OVA-induced mice.

## Methods

### The OVA-induced AR model

**Fig. 1:**
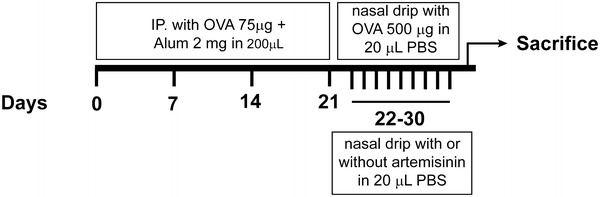
Schematic diagram of OVA-induced allergic rhinitis model. BALB/c mice were sensitized with OVA and aluminum hydroxide on days 0, 7, 14, and 21. All groups except the negative control were sensitized with intranasal instillation with OVA on days 22–30. Artemisinin was nasally administered 1 h before each challenge at 1, 10 or 100 mg/kg on days 22–30. Positive control group was treated with PBS

The AR mouse model was established as previously described [1]. 6-week-old female BALB/c mice, free of murine specific pathogens, were purchased from the Nanjing Model Animal Center. Mice were intraperitoneally injected with 75 µg of OVA and 2 mg of Al(OH)_3_ (Sigma, St. Louis, MO) in 0.2 mL of phosphate buffer solution (PBS) on days 0, 7, 14, and 21. On days 22–30 after initial administration, mice received a nasal drip of OVA 500 µg in 20 µL of PBS, and in normal control were dripped PBS. Artemisinin (Purity ≥ 99.5%), purchased from Aladdin Chemistry Co. Ltd (Shanghai, China), was administered nasally (1, 10 and 100 mg/kg) 1 h before each OVA exposure on days 22–30 (Fig. 1). The control group was treated with PBS. All the mice were maintained in the pathogen-free cage at 22–24 °C and 20% humidity with a 12 h light/dark cycle. Food and water were provided ad libitum and all animal experiments in this study were performed in accordance with the standard guidelines of the Institutional Review Board of Cangzhou Central Hospital.

### Evaluation of induced allergic symptoms

Symptom scores were calculated as previously described [15]. The numbers of sneezing and nose rubbing motions were counted for 15 min after the last allergen exposure and compared to that of the control group.

### Measurement of histamine, OVA-specific IgE and inflammatory factors in mouse serum

Serum levels of histamine, anti-OVA specific IgE and cytokines were measured by ELISA kits (R&D System, Minneapolis, MN) according to the manufacturer’s instruction. Finally, the absorbance at 570 nm (A570) was measured with a multifunction microplate reader, and the concentrations of histamine, anti-OVA specific IgE, TNF, INF-γ, IL-1β and IL-10 were calculated from the standard curve.

### Western blot assay

To analyze the mechanisms of artemisinin in the treatment of allergic rhinitis, Western blot was performed as previously described [16]. 10^7^ Cells were lysed in Nonidet P-40 lysis buffer containing 0.1 mM Na_3_VO_4_ at 4 °C for 30 min. After centrifugation, Cell lysates (100 µg/lane) were electrophoresed on a 10% reducing polyacrylamide gel and transferred onto PVDF membranes (Pharmacia Corporation, NJ, USA). After blocking, the membrane was incubated with goat polyclonal antibodies against JNK (1:1000; Proteintech), ERK (1:5000; Abcam, Cambridge, MA, USA), phos-ERK (1:1000; Cell Signaling Technology, Danvers, MA, USA) and phos-JNK (1:1000; Cell Signaling Technology, USA). The primary antibodies that bound to the target proteins were detected using anti-goat horseradish peroxidase (HRP)-conjugated secondary antibodies (1:3000; Beyotime, China). Protein bands were visualized and analyzed using an enhanced chemiluminescence detection system.

### Flow cytometric analysis of CD4^+^CD25^+^Foxp3^+^ T (Treg) cells

The spleens of the mice in different groups were aseptically removed 24 h after the last challenge. 10^6^ splenic mononuclear cells were stained with fluorescein isothiocyanate- conjugated CD4 Ab (BD Biosciences, Franklin Lakes, NJ, USA) and were incubated with fix/perm solution (eBioscience, San Diego, CA, USA). Fc receptors on cell surfaces were blocked by treatment with excess mouse Fc block (Pharmingen, USA). After the surface staining, cells were stained with PE-labeled FOXP3 Ab (BD Biosciences, USA) and allophycocyanin-CD25 Ab (eBioscience, USA). Treg cells were analyzed by FACSCanto cytometer (BD Bioscience, USA), and data were evaluated with FACSDiva software (BD Bioscience, USA).

### Neurectomy of pterygoid canal

The OVA-induced allergic rhinitis mice were anesthetized. Neurectomy of pterygoid canal was conducted under a microscope. A curved incision was made along the posterior outer edge of the middle turbinate attachment which reached to the sphenoid bone. The operator stripped the mucoperiosteal toward the outside until the exposure of the posterior margin of the sphenopalatine foramen. Subsequently, the vidian nerve was cut and electronically coagulated. After the exposure of the opening of bone-pterygoid canal, the nerves, soft tissue and vessels were coagulated and removed in front of the pterygoid canal. Pterygoid canal were confirmed as description in the literature [17].

### Real time quantitative PCR (RT qPCR)

Total RNA was prepared from local lymph nodes of mice in different groups through the TRIzol Reagent (Invitrogen). Then, RNA was treated by RNase-free DNAse I (Thermo Fisher Scientific) and cDNA was synthesized through the High-Capacity cDNA Reverse Transcription Kit (Thermo Fisher Scientific). SYBR^®^ Premix Ex Taq™ II kit (TaKaRa, Shanghai, China) was employed to examine the mRNA levels of TNF, INF-γ, IL-1β and IL-10 according to the manufacturer’s protocol provided. Relative mRNA levels of these above genes were analyzed via the 2 − ΔΔ*C*t method. All primers were as follows:

TNF

Forward 5′-GGCTTTCCGAATTCACTGGAG-3′

Reverse 5′-CCCCGCCCTTCCAAATAAA-3′

INF-γ

Forward 5′-AGAGCCAGATTATCTCTTTCTACCTCAG-3′

Reverse 5′-CCTTTTTCGCCTTGCTGTTG-3′

IL-1β

Forward 5′-TTGACGGACCCCAAAAGATG-3′

Reverse 5′-AGAAGGTGCTCATGTCCTCA-3′

IL-10

Forward 5′-GACCAGCTGGACAACATACTGCTAA-3′

Reverse 5′-GATAAGGCTTGGCAACCCAAGTAA-3′

GAPDH

Forward 5′-ACC CAG AAG ACT GTG GAC TT-3′

Reverse 5′-TTC TAG ACG GCA GGT CAG GT-3′.

### Statistical analysis

All the experimental data were presented as mean ± SEM of at least three separate tests. In cases of statistical significance, the ranked parameters were compared by one way ANOVA analysis. In all analyses, p > 0.05 indicated no significance, p < 0.05 indicated significance, while p < 0.001 indicated extreme significance.

## Results

### Artemisinin reduces allergic nasal symptoms in OVA-induced mice

**Fig. 2:**
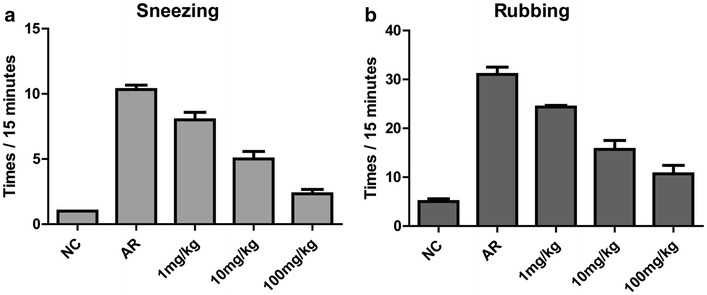
Effects of artemisinin on allergy symptoms in OVA-induced allergic rhinitis mouse model. Total numbers of sneezing (**a**) and nasal rubbing (**b**) motions were counted as description in “Methods”. These two allergy symptoms were significantly inhibited by artemisinin in a dose dependent manner. NC indicated negative control while AR indicated non-treated allergic rhinitis

As shown in Fig. 2a, b, sneezing and nasal rubbing were observed and counted for 15 min after the final nasal challenge, and the mean behavior score was 6.2 ± 1.5 in the NC group, 41.4 ± 3.2 in the AR group, 30.2 ± 2.3 in the 1 mg/kg group, 21.02 ± 1.4 in the 10 mg/kg group and 13.6 ± 1.6 in the 100 mg/kg group, respectively. Wang et al. described that a successful animal model of AR was determined by symptom scores higher than 5 [15]. By day 30, the score was significantly higher in the AR group than that in the NC group (Fig. 2a, b), which met the criterion for successful induction of the AR model. Behavior scores were significantly lower in the 10 and 100 mg/kg artemisinin groups, compared with the AR group (p < 0.001) (Fig. 2a, b). The histological study also showed that artemisinin could reduce the recruitment of inflammatory cells such as eosinophils to the nasal mucosa (Additional file 1: Figure S1A and B).

### Artemisinin reduces the expressions of histamine, OVA-specific IgE and inflammatory factors in AR mouse serum

**Fig. 3:**
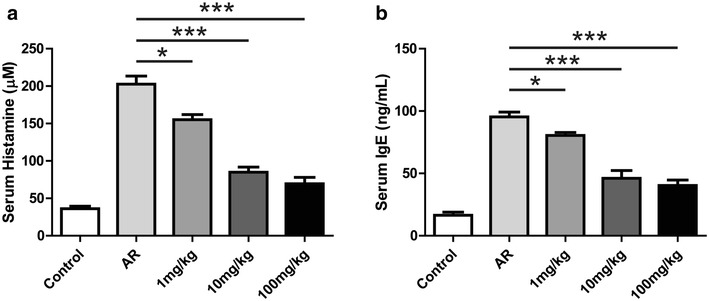
Effects of artemisinin on the levels of histamine and IgE in OVA-sensitized mice. Serum was collected after the sacrifice of the mice. The levels of histamine (**a**) and OVA-specific IgE (**b**) in the serum of different group as indicated were measured by ELISA. *Indicated p < 0.05, ***indicated p < 0.001

**Fig. 4:**
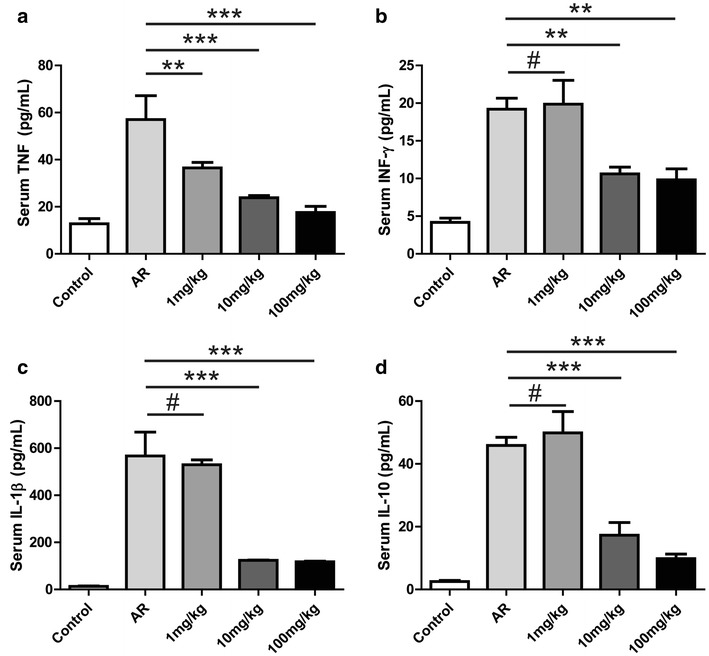
Effects of artemisinin on the levels of inflammatory factors in OVA-sensitized mice. The levels of the inflammatory factors such as TNF (**a**), INF-γ (**b**), IL-1β (**c**) and IL-10 (**d**) were measured in mouse serum by ELISA. ^#^Indicated p > 0.05, **indicated p < 0.01, ***indicated p < 0.001

Histamine and OVA-specific IgE were significantly elevated in the AR group compared with the control group (Fig. 3a, b). Repeated intranasal administration of artemisinin resulted in significantly reduced histamine and OVA-specific IgE antibody levels at 1 mg/kg of artemisinin (p < 0.05) (Fig. 3a, b). At 10 or 100 mg/kg of artemisinin, histamine and IgE levels were extremely lower than the non-treated AR group (p < 0.001) (Fig. 3a, b). The levels of TNF (Fig. 4a), INF-γ (Fig. 4b), IL-1β (Fig. 4c), IL-10 (Fig. 4d), IL-4 (Additional file 1: Figure S2A) and IL-5 (Additional file 1: Figure S2B) in mouse serum displayed significant elevation in the AR group, compared with the control group. At 1 mg/kg of artemisinin, the levels of TNF (Fig. 4a, p < 0.01) and IL-5 (Additional file 1: Figure S2B, p < 0.05) decreased significantly, whereas the levels of INF-γ (Fig. 4b), IL-1β (Fig. 4c), IL-10 (Fig. 4d) and IL-4 (Additional file 1: Figure S2A) did not differ significantly from the AR group (p > 0.05). At 100 mg/kg of artemisinin, IL-4 level decreased significantly compared to the AR group (Additional file 1: Figure S2A, p < 0.05). Moreover, the productions of TNF, INF-γ, IL-1β, IL-10 and IL-5 were significantly inhibited by artemisinin at 100 mg/kg (p < 0.001) (Fig. 4a–d and Additional file 1: Figure S2B). In addition, we detected the mRNA expression levels of TNF, INF-γ, IL-1β and IL-10 in the local lymph nodes by RT-qPCR, and the results were consistent with the serum levels of these cytokines (Additional file 1: Figure S3A–D).

### Artemisinin therapy elevates the percentage of Treg cells

**Fig. 5:**
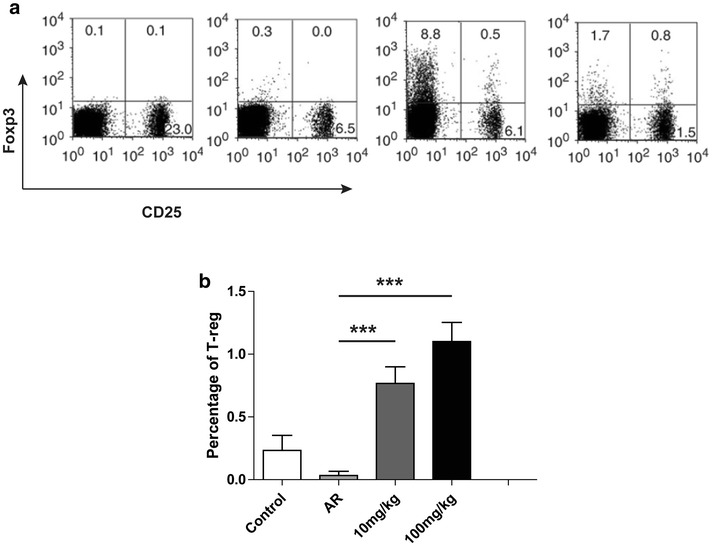
CD4^+^CD25^+^Foxp3^+^ regulatory T (Treg) cells were up-regulated with the treatment of artemisinin. **a** Flow cytometric analysis of CD4^+^CD25^+^Foxp3^+^ T cell subsets. Splenic mononuclear cells were collected from the spleen of the mice in different group and then performed by fluorescence-activated cell sorting analysis. Cells were first gated from CD4 positive portion and then analysis with CD25 and Foxp3. Upper right quadrant represented the CD4^+^CD25^+^Foxp3^+^ T cells, which were regulatory T cells. **b** The statistical analysis of (**a**). ***Indicated p < 0.001

Cells were sorted based on the expression levels of CD25, Foxp3 and CD4. The Treg cells accounted for 0.25 ± 0.13% of all splenic mononuclear cells in the control group (Fig. 5a), while Treg cells were hardly found in the splenic mononuclear cells of OVA-induced mice (Fig. 5a, b). The Treg cell percentage was 0.78 ± 0.12% in the 10 mg/kg group, and 1.08 ± 0.11% in 100 mg/kg group (Fig. 5b). The Treg cell percentages were significantly higher in artemisinin groups than those in the AR group (p < 0.001) (Fig. 5a, b).

### Artemisinin inhibits ERK activation in bone marrow mononuclear cells (BMMCs)

**Fig. 6:**
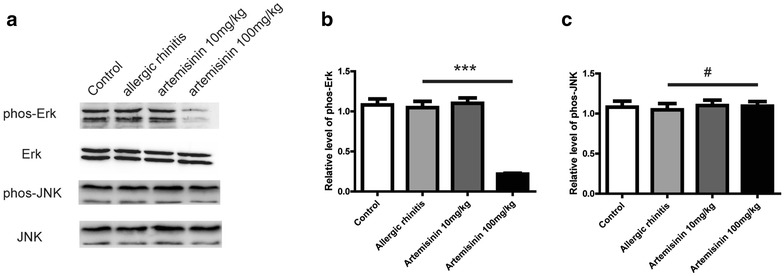
Artemisinin inhibited ERK activation therefore affected the OVA induced allergic rhinitis. **a** Western blot analysis for phosphorylated JNK and phosphorylated ERK in bone marrow mononuclear cells from the mice with or without artemisinin treatment as indicated. **b** and **c** Were the statistical analysis of (**a**). ^#^Indicated p > 0.05, ***indicated p < 0.001

Because the phosphorylation of MAPKs is critical for the allergic responses in AR, we used Western blot to further analyze the mechanisms involved in the anti-allergic effects of artemisinin [18]. Under normal condition, protein levels of ERK, phos-ERK, JNK and phos-JNK in BMMCs were abundant (Fig. 6a). Stimulation of OVA with or without artemisinin treatment had no influence on the protein levels of ERK, JNK and phos-JNK (Fig. 6a–c). The expressions of phos-JNK did not differ significantly in 10 mg/kg group, whereas the protein level of phos-JNK decreased dramatically in 100 mg/kg group (p < 0.001) (Fig. 6a, b). These data suggested that the activation of JNK in AR mouse model was inhibited by the administration of artemisinin.

### Artemisinin and neurectomy of pterygoid canal synergistically alleviate allergy symptoms in OVA-induced mice

**Fig. 7:**
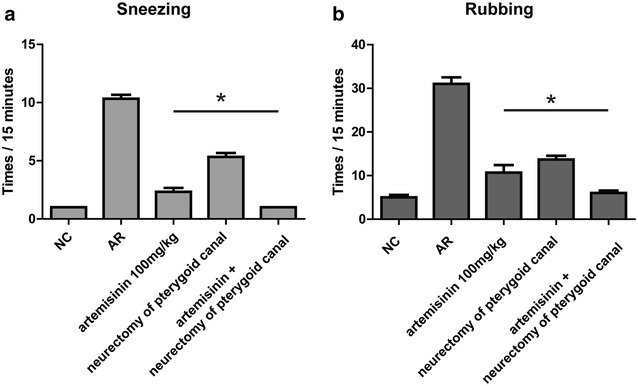
The synergistic effect between artemisinin and neurectomy of pterygoid canal on allergy symptoms in OVA induced allergic rhinitis mouse model. Total number of sneezing (**a**) and nasal rubbing (**b**) motions were significantly inhibited with artemisinin and further inhibited by neurectomy of pterygoid canal. Pterygoid canal were confirmed as description in the literature [17]. *Indicated p < 0.05

To evaluate the effects of combination therapy using 100 mg/kg artemisinin plus neurectomy of pterygoid canal on AR, the animals were randomly assigned into four groups: NC, AR, artemisinin (100 mg/kg) and artemisinin (100 mg/kg) plus neurectomy of pterygoid canal. Behavior scores were significantly higher in the AR group compared with those of the NC group, and artemisinin treatment significantly inhibited the OVA-induced increase of behavioral scores. More surprisingly, artemisinin plus neurectomy of pterygoid canal showed better efficacy than artemisinin alone and almost abolished the increase of behavioral scores in OVA-induced mice (p < 0.05) (Fig. 7a, b).

## Discussion

Artemisinin, a sesquiterpene lactone, is isolated from the traditional Chinese herb *Artemisia annua* L. [19]. It was discovered by Chinese scientist Tu Youyou, who has won the 2015 Nobel Prize in Medicine for her discovery. Artemisinin combination therapies (ACTs) are now standard treatment worldwide for malaria [20]. Numerous studies have demonstrated the biological actions of artemisinin in various diseases [9–13]. Although artemisinin has anti-inflammatory effect, its anti-allergic activity has not been commonly reported yet. In the current study, we used a mouse model of AR to examine the effect of artemisinin intranasal instillation on allergic symptoms and explore the underlying mechanisms.

Allergic rhinitis, characterized by sneezing, nasal itching and rhinorrhea, is very common. The OVA-induced AR model displayed characteristic allergic behaviors, such as increased sneezing and increased nose rubbing motions. The administration of artemisinin decreased the allergic symptom score in a dose-dependent manner. AR is an IgE-mediated inflammatory disorder induced after exposure to environmental allergens. The activated inflammatory cells accumulate in the nasal mucosa and release allergic mediators, such as histamine and inflammatory factors, to maintain inflammatory reactions. Previous studies reported that anti-allergic effects of natural anti-allergic substances, such as bee venom, piperine and curcumin, might be used to inhibit the production of allergic mediators [1, 5, 21]. In this study, AR mouse model displayed increased levels of histamine, IgE and inflammatory factors (TNF, INF-γ, IL-1β, IL-10, IL-4 and IL-5) in the serum. Moreover, the mRNA levels of inflammatory factors (TNF, INF-γ, IL-1β and IL-10) were also significantly increased in the local lymph nodes of AR mice. Interestingly, treatment with artemisinin inhibited the release of allergic mediators. These above molecular changes were supported by the allergy symptom relief of AR mice after the intranasal instillation of artemisinin.

Many studies have shown that the MAPK signaling pathways play important roles in the production and release of inflammatory mediators by activated inflammatory cells in allergic inflammation [22, 23]. Therefore, to examine the effect of artemisinin on the MAPK signaling pathways, the total and phosphorylated levels of ERK and JNK in BMMCs from the mice with or without artemisinin treatment were analyzed by Western blot. The Western bolt results showed that 100 mg/kg of artemisinin significantly suppressed the phosphorylation of ERK, while the levels of ERK, JNK and p-JNK remained the same. These findings indicated the potential role of MAPK pathways in the suppression of inflammatory mediators by artemisinin in OVA-induced allergic inflammation. In other words, one possible explanation for the anti-inflammatory effects of artemisinin might be the inhibition of MAPK pathways, which reduced the levels of inflammatory mediators. It is also well-known that MAPKs play important roles in the activation of NF-κB, which regulates a wide variety of genes involved in the allergy process [24, 25]. Our study illustrated that artemisinin inhibited the activation of ERK, while the effect of artemisinin on the NF-κB pathway should be validated in future studies.

CD4^+^CD25^+^Foxp3^+^ regulatory T (Treg) cells are a small T cell population, which are instrumental in modulating the immune system and maintaining tolerance to self-antigens. There are increasing evidences suggesting that Treg cells can inhibit the activation of numerous immune cells and curtail aberrant immune activation [26, 27]. In our study, flow cytometry analysis showed that the percentages of Treg cells were increased significantly in the artemisinin treatment groups as compared to the AR group. These results indicated that the anti-allergic effect of artemisinin might also be related to the increased Treg cells. In addition, Coleman et al. reported that CD4^+^Foxp3^+^CD25^−^ cells were the dominant Treg population in the lungs of mice [28]. In Fig. 5a, flow cytometry data showed that artemisinin at 10 mg/kg also induced the population of CD4^+^Foxp3^+^CD25^−^ cells, consistent with previous report [28]. It is well-known that Treg cells are the main producers of IL-10, however we found that artemisinin induced more Treg whereas at the same time reduced IL-10 expression in mouse serum. Hence, artemisinin administration may also influence the Treg cell production in local lymph node of AR mice, which should be further analyzed in the future.

Daoud et al. demonstrated that some allergic mediators of AR might stimulate the nervous system, and the neural functions interacted with the immune system, subsequently influencing the inflammatory process [4]. Neurectomy of pterygoid canal has been proved to be effective in the management of AR [29, 30]. Thus, we performed artemisinin plus neurectomy of pterygoid in the AR mouse model to identify whether artemisinin combination therapy could display a synergistic anti-allergic effect. Much to our delight, the results of allergic symptoms evaluation showed that artemisinin combined with neurectomy of pterygoid exhibited much better efficacy than artemisinin alone, and almost abolished the increased allergic symptom scores in AR mice, which supported our above hypothesis.

In summary, the above results from the current study illustrated that artemisinin exhibited anti-allergic effect in the AR animal model, which extended previous reports demonstrating the therapeutic effects of artemisinin on various diseases. We also elucidated that artemisinin exerted anti-allergic role in OVA-induced AR mice by reducing the level of allergic mediators, along with the reduced phosphorylation of ERK and the induction of Treg cells. Thus, the administration of artemisinin may provide a novel therapeutic strategy for AR treatment. Meanwhile, the synergistic effect of artemisinin combination therapy also suggested that artemisinin combined with neurectomy of pterygoid canal was a much more promising strategy to inhibit inflammation in AR, and might be utilized in the clinic.

## Conclusions

Our present study provides solid evidence that artemisinin exhibits anti-allergic roles by suppressing allergic responses in AR mouse model. The possible mechanism is that artemisinin treatment decreases expressions of allergic mediators in AR by inhibiting ERK activation and increasing Treg cell population. Moreover, artemisinin combined with neurectomy of pterygoid, a more promising strategy, exhibits better efficacy than artemisinin alone.

## Additional file

**Additional file 1: Figure S1.** Infiltration of eosinophils (Red arrows) in nasal mucosa (under 100 times). (A) Representative histochemical photographs of the mice with different treatments as indicated. (B) Statistical histogram of panel A. #Indicated p>0.05, *indicated p<0.05. **Figure S2.** Effects of artemisinin on the serum levels of IL-4 and IL-5 in OVA-sensitized mice. Serum was collected after the sacrifice of the mice. The levels of IL-4 (A) and IL-5 (B) in the serum of different group as indicated were measured by ELISA. #Indicated p>0.05, *indicated p<0.05, ***indicated p<0.001. **Figure S3.** Effects of artemisinin on the mRNA levels of inflammatory factors in local lymph nodes. The levels mRNA of the inflammatory factors such as TNF-α (A), INF-γ (B), IL-1β (C) and IL-10 (D) in local lymph nodes were measured by RT-qPCR in the mice with different treatments as indicated. #Indicated p>0.05, *indicated p<0.05, **indicated p<0.01.

## Abbreviations

AR: allergic rhinitis
OVA: ovalbumin
IgE: immunoglobulin E
Treg: CD4^+^CD25^+^Foxp3^+^ T

## References

[1] Shin SH, Kim YH, Kim JK, Park KK (2014). Anti-allergic effect of bee venom in an allergic rhinitis mouse model. Biol Pharm Bull.

[2] Kianmehr M, Haghmorad D, Nosratabadi R, Rezaei A, Alavinezhad A, Boskabady MH (2017). The Effect of *Zataria multiflora* on Th1/Th2 and Th17/T Regulatory in a Mouse Model of Allergic Asthma. Front Pharmacol.

[3] Hong SH, Kim SR, Choi HS, Ku JM, Seo HS, Shin YC, Ko SG (2014). Effects of Hyeonggaeyeongyo-tang in ovalbumin-induced allergic rhinitis model. Mediators Inflamm.

[4] Daoud A, Xie Z, Ma Y, Wang T, Tan G (2014). Changes of T-helper type 1/2 cell balance by anticholinergic treatment in allergic mice. Ann Allergy Asthma Immunol.

[5] Aswar U, Shintre S, Chepurwar S, Aswar M (2015). Antiallergic effect of piperine on ovalbumin-induced allergic rhinitis in mice. Pharm Biol.

[6] Oh HA, Kim MJ, Shin TY, Kim HM, Jeong HJ (2014). The antiallergic mechanisms of *Citrus sunki* and bamboo salt (K-ALL) in an allergic rhinitis model. Exp Biol Med (Maywood).

[7] Daily JP (2017). Malaria 2017: update on the clinical literature and management. Curr Infect Dis Rep.

[8] Guo S, Kyaw MP, He L, Min M, Ning X, Zhang W, Wang B, Cui L (2017). Quality testing of artemisinin-based antimalarial drugs in Myanmar. Am J Trop Med Hyg.

[9] Efferth T, Dunstan H, Sauerbrey A, Miyachi H, Chitambar CR (2001). The anti-malarial artesunate is also active against cancer. Int J Oncol.

[10] Wang J, Zhang J, Shi Y, Xu C, Zhang C, Wong YK, Lee YM, Krishna S, He Y, Lim TK (2017). Mechanistic investigation of the specific anticancer property of artemisinin and its combination with aminolevulinic acid for enhanced anticolorectal cancer activity. ACS Cent Sci.

[11] Singh NP, Lai H (2001). Selective toxicity of dihydroartemisinin and holotransferrin toward human breast cancer cells. Life Sci.

[12] Singh NP, Lai HC (2004). Artemisinin induces apoptosis in human cancer cells. Anticancer Res.

[13] Wong HR, Menendez IY (1999). Sesquiterpene lactones inhibit inducible nitric oxide synthase gene expression in cultured rat aortic smooth muscle cells. Biochem Biophys Res Commun.

[14] Zhao X, Wang L, Zhang H, Zhang D, Zhang Z, Zhang J (2017). Protective effect of artemisinin on chronic alcohol induced-liver damage in mice. Environ Toxicol Pharmacol.

[15] Wang W, Zhu Z, Zhu B, Ma Z (2011). Peroxisome proliferator-activated receptor-gamma agonist induces regulatory T cells in a murine model of allergic rhinitis. Otolaryngol Head Neck Surg.

[16] Uemura Y, Liu TY, Narita Y, Suzuki M, Matsushita S (2008). 17 Beta-estradiol (E2) plus tumor necrosis factor-alpha induces a distorted maturation of human monocyte-derived dendritic cells and promotes their capacity to initiate T-helper 2 responses. Hum Immunol.

[17] Shimozawa A (1973). An electron microscopic analysis of the nerve of the pterygoid canal in the mouse. Anat Rec.

[18] Zhang N, Li H, Jia J, He M (2015). Anti-inflammatory effect of curcumin on mast cell-mediated allergic responses in ovalbumin-induced allergic rhinitis mouse. Cell Immunol.

[19] White NJ (1997). Assessment of the pharmacodynamic properties of antimalarial drugs in vivo. Antimicrob Agents Chemother.

[20] Lv Z, Zhang L, Tang K (2017). New insights into artemisinin regulation. Plant Signal Behav.

[21] Llosa NJ, Cruise M, Tam A, Wicks EC, Hechenbleikner EM, Taube JM, Blosser RL, Fan HN, Wang H, Luber BS (2015). The vigorous immune microenvironment of microsatellite instable colon cancer is balanced by multiple counter-inhibitory checkpoints. Cancer Discov.

[22] Cho MS, Park WS, Jung WK, Qian ZJ, Lee DS, Choi JS, Lee DY, Park SG, Seo SK, Kim HJ (2014). Caffeic acid phenethyl ester promotes anti-inflammatory effects by inhibiting MAPK and NF-kappaB signaling in activated HMC-1 human mast cells. Pharm Biol.

[23] Won Jung H, Jung JK, Weon Cho C, Kang JS, Park YK (2012). Antiallergic effect of KOB03, a polyherbal medicine, on mast cell-mediated allergic responses in ovalbumin-induced allergic rhinitis mouse and human mast cells. J Ethnopharmacol.

[24] Zhou YJ, Wang H, Sui HH, Li L, Zhou CL, Huang JJ (2016). Inhibitory effect of baicalin on allergic response in ovalbumin-induced allergic rhinitis guinea pigs and lipopolysaccharide-stimulated human mast cells. Inflamm Res.

[25] Zhang D, Wang J, Li Z, Zhou M, Chen Q, Zeng X, Chen Y (2015). The activation of NF-kappaB in infiltrated mononuclear cells negatively correlates with treg cell frequency in oral lichen planus. Inflammation.

[26] Brusko TM, Putnam AL, Bluestone JA (2008). Human regulatory T cells: role in autoimmune disease and therapeutic opportunities. Immunol Rev.

[27] Chen W (2011). Tregs in immunotherapy: opportunities and challenges. Immunotherapy.

[28] Coleman MM, Finlay CM, Moran B, Keane J, Dunne PJ, Mills KH (2012). The immunoregulatory role of CD4(+) FoxP3(+) CD25(−) regulatory T cells in lungs of mice infected with *Bordetella pertussis*. FEMS Immunol Med Microbiol.

[29] Konno A, Togawa K (1979). Vidian neurectomy for allergic rhinitis. evaluation of long-term results and some problems concerning operative therapy. Arch Otorhinolaryngol.

[30] Tan G, Ma Y, Li H, Li W, Wang J (2012). Long-term results of bilateral endoscopic vidian neurectomy in the management of moderate to severe persistent allergic rhinitis. Arch Otolaryngol Head Neck Surg.

