# Supplementary material for: Effect of artemisinin and neurectomy of pterygoid canal in ovalbumin-induced allergic rhinitis mouse model
Source: Allergy Asthma Clin Immunol. 2018 Jun 11;14:22. doi: 10.1186/s13223-018-0249-6 (PMC5994650; doi:10.1186/s13223-018-0249-6)
Supplement: Supplementary file 1 — Additional file 1: Figure S1. Infiltration of eosinophils (Red arrows) in nasal mucosa (under 100 times). (A) Representative histochemical photographs of the mice with different treatments as indicated. (B) Statistical histogram of panel A. #Indicated p>0.05, *indicated p<0.05. Figure S2. Effects of artemisinin on the serum levels of IL-4 and IL-5 in OVA-sensitized mice. Serum was collected after the sacrifice of the mice. The levels of IL-4 (A) and IL-5 (B) in the serum of different group as indicated were measured by ELISA. #Indicated p>0.05, *indicated p<0.05, ***indicated p<0.001. Figure S3. Effects of artemisinin on the mRNA levels of inflammatory factors in local lymph nodes. The levels mRNA of the inflammatory factors such as TNF-α (A), INF-γ (B), IL-1β (C) and IL-10 (D) in local lymph nodes were measured by RT-qPCR in the mice with different treatments as indicated. #Indicated p>0.05, *indicated p<0.05, **indicated p<0.01. [file 13223_2018_249_MOESM1_ESM.docx]

**Additional file**

**
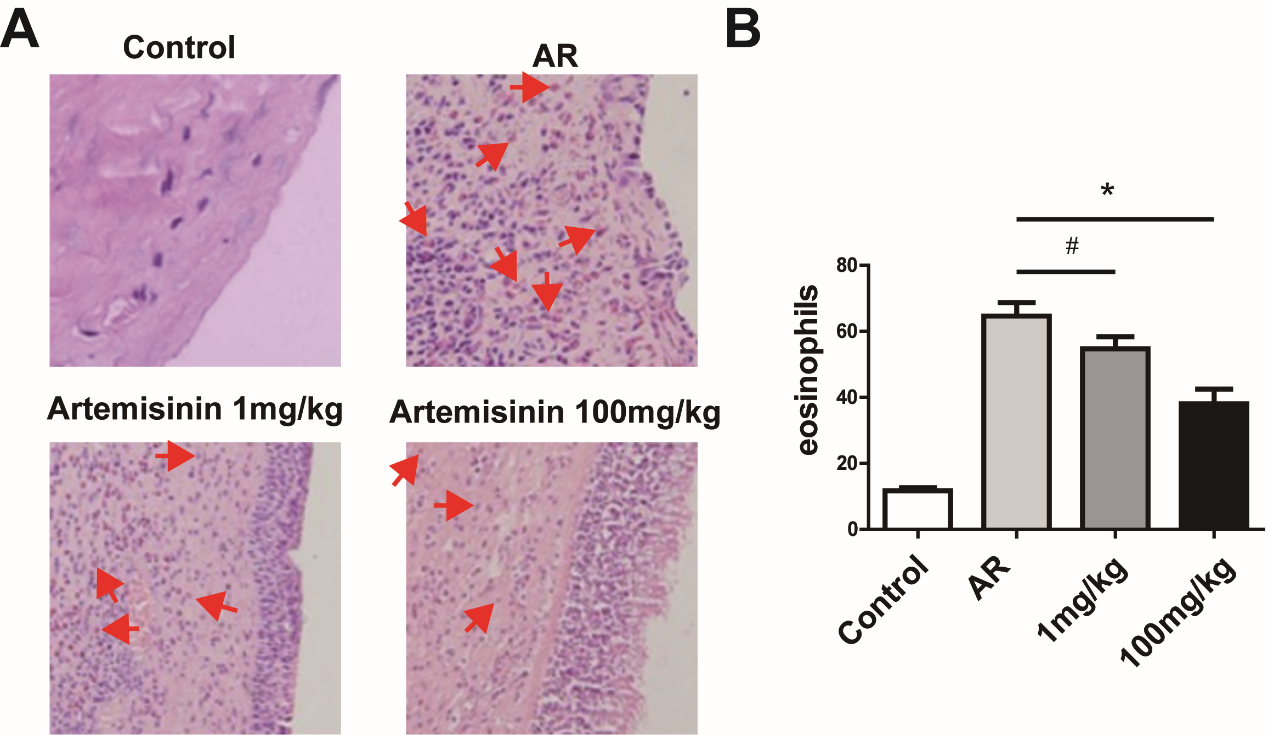
**

**Figure S1. Inﬁltration of eosinophils (Red arrows) in nasal mucosa (under 100 times).** (A) Representative histochemical photographs of the mice with different treatments as indicated. (B) Statistical histogram of panel A. # indicated p>0.05, * indicated p<0.05.

**
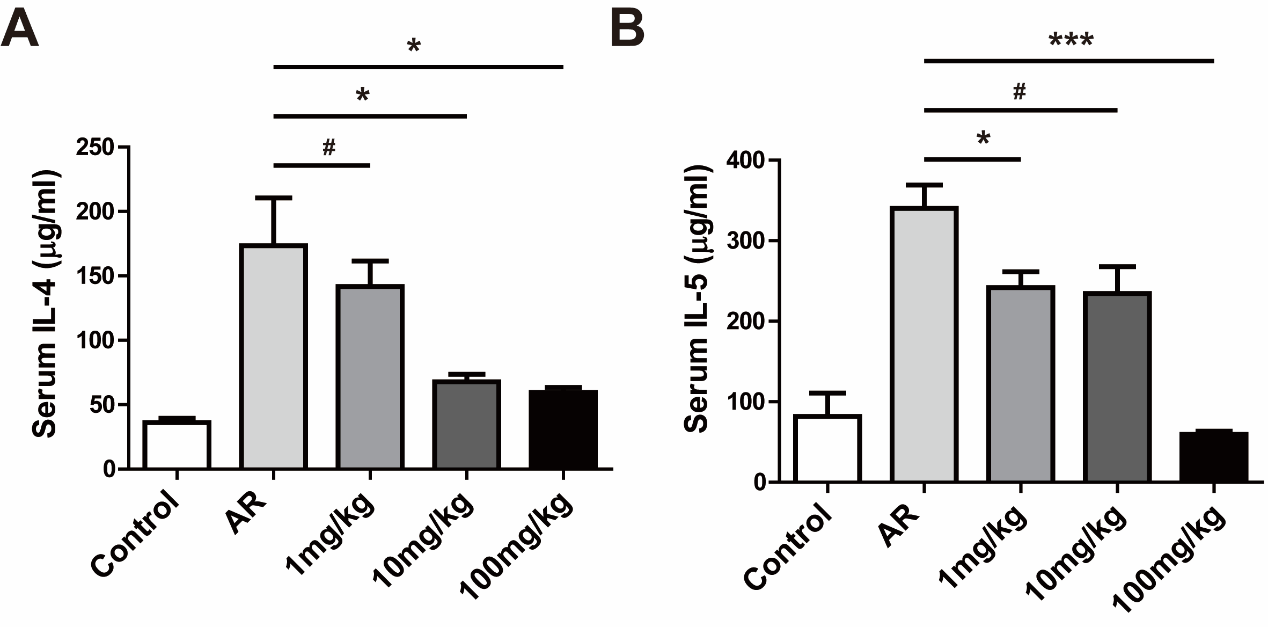
**

**Figure S2. Effects of artemisinin on the serum levels of IL-4 and IL-5 in OVA-sensitized mice.** Serum was collected after the sacrifice of the mice. The levels of IL-4 (A) and IL-5 (B) in the serum of different group as indicated were measured by ELISA. # indicated p>0.05, * indicated p<0.05, *** indicated p<0.001.

**
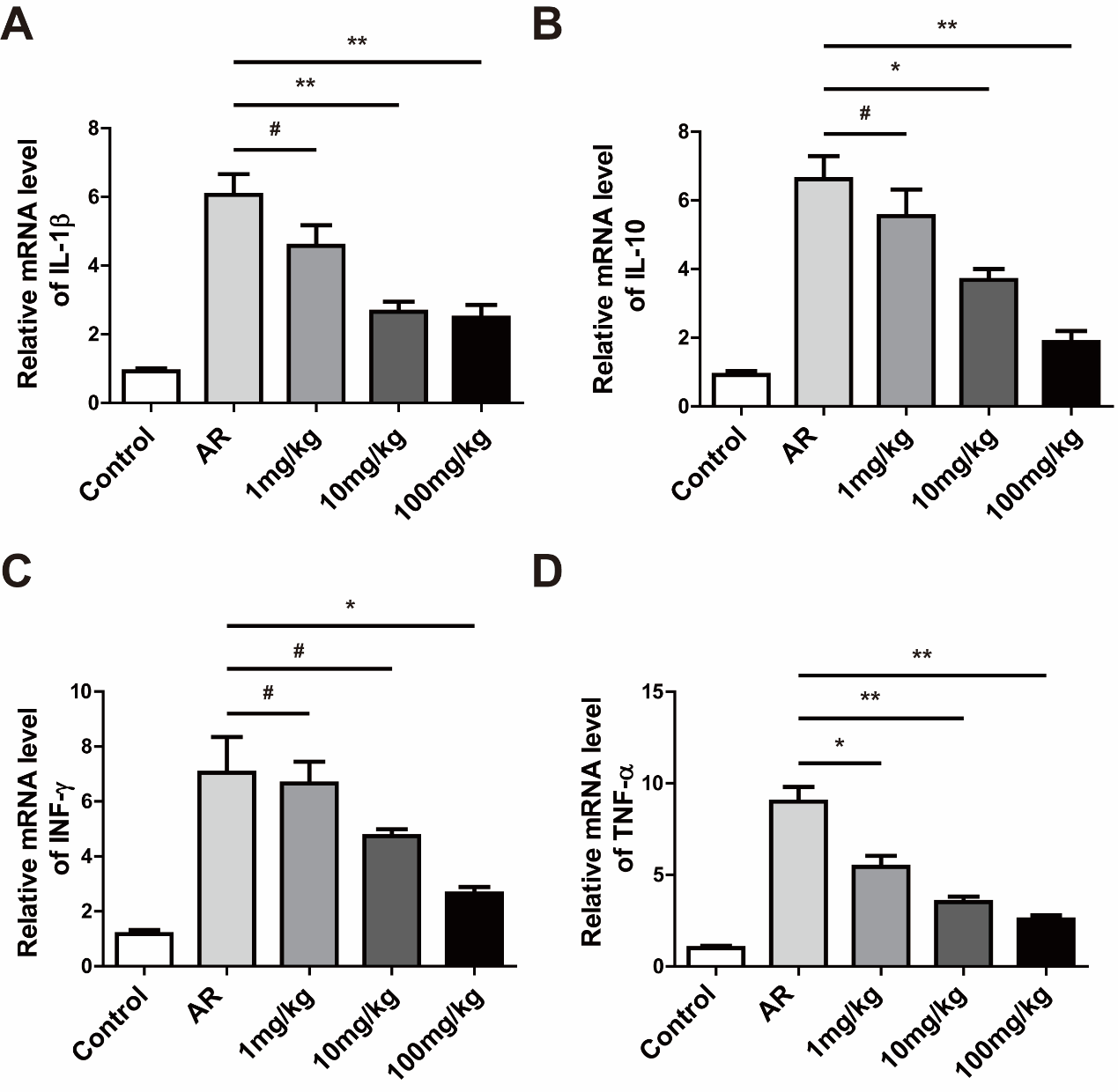
**

**Figure S3. Effects of artemisinin on the mRNA levels of inflammatory factors in local lymph nodes.** The levels mRNA of the inflammatory factors such as TNF-α (A), INF-γ (B), IL-1β (C) and IL-10 (D) in local lymph nodes were measured by RT-qPCR in the mice with different treatments as indicated. # indicated p>0.05, * indicated p<0.05, ** indicated p<0.01.
